# Neuroprotective Effect of Ropinirole Lipid Nanoparticles Enriched Hydrogel for Parkinson’s Disease: In Vitro, Ex Vivo, Pharmacokinetic and Pharmacodynamic Evaluation

**DOI:** 10.3390/pharmaceutics12050448

**Published:** 2020-05-13

**Authors:** Narendar Dudhipala, Thirupathi Gorre

**Affiliations:** 1 Department of Pharmaceutics, Vaagdevi Pharmacy College, Warangal 506 005, Telangana State, India; 2 Department of Pharmacology and Clinical Pharmacy, Vaagdevi Institute of Pharmaceutical Sciences, Warangal 506 005, Telangana State, India; tiru54@gmail.com

**Keywords:** Parkinson’s disease, ropinirole, lipid nanoparticles, hydrogel, ex vivo, pharmacokinetics, pharmacodynamics

## Abstract

Parkinson’s disease (rp) is a progressive neurodegenerative disorder. Ropinirole (RP) is a newer generation dopamine agonist used for the treatment of PD. It is prescribed as oral dosage form. However, limited oral bioavailability and frequent dosing limits the RP usage. The objective of the current investigation was to develop, optimize, evaluate pharmacokinetic (PK) and pharmacodynamic (PCD) activity of RP loaded solid lipid nanoparticles (RP-SLNs) and nanostructured lipid carriers (RP-NLCs) and containing hydrogel (RP-SLN-C and RP-NLC-C) formulations for improved oral and topical delivery. RP loaded lipid nanoparticles were optimized and converted to hydrogel using carbopol 934 as the gelling polymer. PK and PCD studies in haloperidol-induced PD were conducted in male Wistar rats. In vitro and ex vivo permeation studies showed sustained release profile and enhanced permeation compared with control formulations. Differential scanning calorimeter and X-ray diffraction studies revealed amorphous transformation; scanning electron microscope showed the spherical shape of RP in lipid nanoparticles. PK studies showed 2.1 and 2.7-folds enhancement from RP-SLN and RP-NLC from oral administration, 3.0 and 3.3-folds enhancement from RP-SLN-C and RP-NLC-C topical administration, compared with control formulations, respectively. RP-SLN-C and RP-NLC-C showed 1.4 and 1.2-folds topical bioavailability enhancement compared with RP-SLN and RP-NLC oral administration, respectively. PCD studies showed enhanced dopamine, glutathione, catalase levels and reduced lipid peroxidation levels, compared with the haloperidol-induced PD model. Overall, the results demonstrated that lipid nanoparticles and corresponding hydrogel formulations can be considered as an alternative delivery approach for the improved oral and topical delivery of RP for the effective treatment of PD.

## 1. Introduction

Parkinson’s disease (PD), is a progressive neurological brain disorder that affects muscle stiffness and movements. The condition is usually gradual, with symptoms becoming more severe over time [1]. PD affects approximately seven million people globally and one million people in the united states. PD is more common in the elderly and prevalence rises from 1% in those over 50 years of age to 4% of the population over 80 [2]. PD is characterized by tremor, rigidity, postural abnormalities, stooped posture, bradykinesia, akinesia and festinating gait [3]. The significant pathological change in patients with PD is the loss of melanin-containing dopaminergic neurons in zonacompacta of the substantianigra [4]. These pigmented neurons have been identified as nigrostriatal dopamine neurons; loss of these neurons results in a decrease of dopamine (DA) content in the striatum [5].

Ropinirole (RP), a new non-ergoline D2/D3 dopamine receptor agonist, binds specifically to D2-receptors in striatum and substantia nigra, with selectivity similar to that of dopamine. It is supposed to inducement its antiparkinsonian action via enhancing striatal neuronal dismissal rates, through the selective stimulation of D2-dopamine receptors. RP has poor oral bioavailability (50%), due to extensive hepatic first-pass metabolism with an elimination half-life of 5.8 h and *t*_max_ of below one hour. Currently, RP conventional tablet dosage forms with 3–9 mg dose are available on the market, which is unable to attain adequate oral bioavailability. IV administration is very irritating and is not advised [6,7]. Therefore, alternative routes and drug delivery systems are needed to improve its therapeutic efficacy. Further, RP is effective both as monotherapy as well as combination therapy with levodopa, whereby it helps in reduction of the dose of levodopa. The continuous delivery of RP from the transdermal system may delay or prevent the onset of levodopa-related motor complications due to continuous dopaminergic stimulation [8]. It could be anticipated that a once-daily regimen shall significantly increase patient compliance (while at the same time reducing the burden of the caregiver [9]. Therefore, in the current investigation transdermal route was selected as an alternative prime of the route of administration for RP.

The transdermal route has been investigated for several drugs in the past few decades. However, its use is limited due to the understandably low permeability of the skin to many drugs [10]. The primary issue regarding the transdermal route is the anatomical organization of skin. For many years, drugs have been applied for their local effect on the skin [8,9,11]. Recently, drugs have been shown to achieve better therapeutic outcomes through the transdermal route than by oral administration. Various approaches have been used to alter skin to enhance the transport of therapeutic agents, such as using penetration enhancers [12], iontophoresis [13], microneedles [14] and lipid vesicle carriers [15]. The vesicular carrier systems used for topical application of bioactive include lipid nanoparticles such as solid lipid nanoparticles (SLNs) [16], and nanostructured lipid carriers (NLCs) [17], transfersomes [18], liposomes, niosomes and ethosomes [15,19] have been investigated.

SLNs and NLCs dispersed in hydrogels are considered as an alternative and unique delivery system for enhanced dermal applications than traditional topical and dermatological formulation. In general, SLNs are submicron nanoformulations with 50–1000 nm size, made of solid lipids with surfactants as stabilizing agents, and the particles remain in the solid-state [20,21]. NLCs are the second generation of SLNs, in which one part of the solid lipid has been replaced with liquid lipid [22]. Both SLNs and NLCs could combine advantages such as controlled release, large scale production and toxicologically acceptable compared with other colloidal carrier systems. Enhancement of oral bioavailability and pharmacodynamic activity of entrapped drugs via the modification of dissolution rate [23,24] and the targeting of drugs were reported, by using SLNs and NLCs in various application routes [25].

SLNs and NLCs are applied to transdermal and dermatological applications due to their various desirable effects on skin besides the characteristics of a colloidal carrier system. They are well suited for use on damaged or inflamed skin because they are prepared with non-irritant and non-toxic lipids. Furthermore, these lipid nanocarriers showed occlusive properties as a result of film formation on the skin, which reduces transdermal water loss and also favored drug penetration into the skin [23,24,25,26]. Previously, the application of SLN and NLC containing hydrogels for the enhanced transdermal delivery of various drugs such as triptolide [27], flurbiprofen [28] and RP [29] was reported.

The objective of the present investigation was to design, development and optimization of RP loaded SLN (RP-SLN) and NLC (RP-NLC) formulation and corresponding hydrogel (RP-SLN-C and RP-NLC-C) for enhanced neuroprotective activity in the treatment of PD. Hence, RP-SLN and RP-NLC formulations were prepared using a known established method and optimized based on physicochemical characteristics and converted to RP-SLN-C and RP-NLC-C using carbopol 934 as a gelling polymer. Furthermore, lipid nanoparticle and hydrogel formulations were evaluated for in vitro release, ex vivo permeation, pharmacokinetic (PK) and pharmacodynamic (PCD) activity in haloperidol-induced PD male Wistar rats, in vivo, comparison with RP hydrogel (RP-C) and RP suspension (RP-S) as control formulations.

## 2. Materials

Ropinirole was a gift sample from Wockhardt Pharmaceuticals Pvt. Ltd., Aurangabad, India. Propylene glycol monocaprylate (Capryol^®^ 90), tripalmitin (Dyanasn ™114), polyoxyethylene sorbitan monolaurate (Tween^®^ 20), haloperidol, glutathione reductase (GSH), 5-5-dithio-bis-2- nitrobenzoic acid (DTNB), thiobarbituric acid (TBA), ethylene-diamine tetraacetic acid (EDTA) and Centrisart filters (10 kDa molecular weight) were purchased from Sigma Aldrich Chemicals Pvt Ltd., Bangalore, India. Carbopol 934 was procured from Hi-Media, Mumbai, India. High-performance liquid chromatography (HPLC) grade acetonitrile and ammonium acetate were procured from Merck Hyderabad, India. Water was obtained from Milli-Q water purification system (Millipore, MA, USA). All other chemicals and solvents were of analytical grade.

### 2.1. Animals

Male albino Wistar rats (180–210 g) were used to study the ex vivo skin permeation, skin irritation, PK and PCD activity in the haloperidol induced PD model. The animals were procured from Mahaveer Enterprises, Hyderabad, India. They are housed into a group of six rats per cage and maintained at 25 ± 1 °C, with relative humidity 50 ± 5% and 12:12 h dark/light cycle. The animals had free access to food (standard chew pellets) and water ad libitum. The institutional animal ethics committee (1047/ac/o7/CPCSEA, dated 24-07-2016; amendment dt: 25-04-2018) approved all the experimental procedures.

### 2.2. HPLC Method for In Vitro Studies

The RP content from formulations, in vitro release and ex vivo permeation studies, was estimated by using the slightly modified previously published HPLC method [30]. HPLC separation was achieved using a Phenomenex C_18_ Luna column (250 mm × 4 mm, 5 µ), with a mobile phase composed of acetonitrile-phosphate buffer-triethanolamine (58:42:0.3, *v/v*) adjusted at pH 6.0 at a flow rate 1.0 mL/min an AUFS of 1.0000. The UV detector was operated at 245 nm. The method was linear within the range 2.5–50 µg/mL; the limits of detection (LOD) and quantitation (LOQ) found were 1.0 µg mL and 2.5 µg/mL.

### 2.3. Preparation of RP Loaded Lipid Nanocarriers

RP-SLN and RP-NLC formulation was yielded by hot melt emulsification coupled with ultra-wave sonication method [31,32]. The composition of the optimized RP-SLN and RP-NLC formulation is presented in Table 1. Briefly, solid lipid, RP and solid lipid, liquid lipid, RP as lipid phase for RP-SLN and RP-NLC, respectively, was heated at temperature 65 ± 5 °C in a hot water bath (above the 5 °C of solid lipid melting point). The aqueous phase containing Poloxamer 188 (1.0% *w/v*) was dissolved in bi-distilled MilliQ water and heated at the same temperature as the liquid phase. The hot aqueous phase was added to the hot liquid phase under constant stirring at 2000 rpm. The premix was subjected to homogenization using a homogenizer (Diax900, Heidolph, Germany) at 12,000 rpm for 10 min. The hot coarse emulsion was cooled to room temperature and subjected to probe sonication using12T probe sonicator (Vibracell, Sonics, Newtown, CT, USA) for 15 min at 40% amplitude in an ice immersed beaker. The formed dispersion was allowed to be at room temperature and yielded the RP-SLN and RP-NLC formulations. The final concentration of RP was maintained at 2 mg/mL.

### 2.4. Preparation of RP Suspension (RP-S)

The RP loaded suspension (RP-S) formulation used was prepared by dissolving 0.2% *w/v* of RP in Tween ^®^ 20 (1% *w/v*) as a suspending agent and stirred at 2000 pm for 1 h at room temperature. The total volume of the preparation was 10 mL. This formulation was used as a control for comparison studies of RP-SLN and RP-NLC formulations.

### 2.5. Preparation of RP-SLN-C and RP-NLC-C

Hydrogel formulations of RP-SLN and RP-NLC were prepared by using 1% *w/v* of carbopol 934 as a gelling agent. Initially, the aqueous phase of a gelling agent was prepared by dispersing the carbopol in double distilled water containing glycerol (10% *w/v*) and soaking overnight for swelling. RP-SLN and RP-NLC formulation and gelling hydrogel were mixed in a high-speed stirrer (Remi, Mumbai, India), at approximately 100 rpm for 5 min, to yield RP-SLN-C and RP-NLC-C gels, containing a final concentration equal to 0.2% *w/v* of RP. Triethanolamine (0.3% *w/v*) was used to neutralize the pH of the formed hydrogel formulations for topical application range. The formed RP-SLN-C and RP-NLC-C formulation were left overnight to remove any entrapped air. Similarly, the conventional RP hydrogel (RP-C) formulation was also prepared with the same concertation of drug loading. The composition of lipid nanoparticle and conventional hydrogel formulation was shown in Table 1.

### 2.6. Characterization of RP-SLN and RP-NLC

#### 2.6.1. Particle Size, Polydispersity Index (PDI) and Zeta Potential (ZP)

The particle size, PDI and ZP of RP-SLN and RP-NLC formulation was analyzed, using a photon correlation spectroscopy with Zetasizer (Nano ZS90, Malvern, UK). For the measurement of particle size and PDI, the prepared lipid nanoformulations were diluted (50 times) with double distilled water, to get optimum kilo counts per second for the measurements. ZP of the formulations was measured with the same diluted sample, using an electrode cell based on Smoluchowski equation. All measurements were carried out at 25 °C and in triplicate [33].

#### 2.6.2. Total Drug Content and Drug Loading (DL)

For drug content and DL, about 100 µL of the RP-SLN and RP-NLC formulation was dissolved in 900 µL of ethanol and vortex for 5 min at 5000 rpm. Then, further dilutions were made with the mobile phase [34]. The diluted samples were injected onto the column of HPLC. The amount of RP in lipid nanoformulations was estimated. Similarly, the DL of RP in lipid nanoparticles was estimated by using the following Equation (1).

(1) $$DL\;\left(\%\right)=\left[\frac{\mathrm{Total}\;\mathrm{amount}\;\mathrm{of}\;\mathrm{RP}-\mathrm{amount}\;\mathrm{of}\;\mathrm{unentrapped}\;\mathrm{RP}}{\mathrm{Total}\;\mathrm{weight}\;\mathrm{of}\;\mathrm{the}\;\mathrm{lipids}}\right]\times 100$$

#### 2.6.3. Entrapment Efficiency (EE)

EE of RP-SLN and RP-NLC formulation was determined by measuring the concentration of the unentrapped (free drug) drug in an aqueous medium, as per the earlier reported method [35]. The aqueous medium was separated by ultra-filtration using centrisart tubes (Sartorius, Goettingen, Germany), which consisted of a filter membrane (M.Wt. cut off 20 kDa) at the base of the sample recovery chamber. About 2.5 mL of the SLN and NLC formulation was placed in the outer chamber, and the sample recovery chamber was placed on top of the sample and centrifuged (Remi, India) at 15,000 rpm for 15 min. The filtrate was further diluted with ethanol and analyzed by the HPLC method. The EE of the nanoformulations was calculated using the following Equation (2):

(2) $$\mathrm{EE}\;\left(\%\right)=\left[\frac{\mathrm{Total}\;\mathrm{amount}\;\mathrm{of}\;\mathrm{RP}\;\mathrm{in}\;\mathrm{formulation}-\mathrm{Amount}\;\mathrm{of}\;\mathrm{free}\;\mathrm{RP}}{\mathrm{The}\;\mathrm{total}\;\mathrm{amount}\;\mathrm{of}\;\mathrm{RP}\;\mathrm{in}\;\mathrm{the}\;\mathrm{formulation}}\right]\times 100$$

### 2.7. Evaluation of RP-SLN-C and RP-NLC-C

#### 2.7.1. Visual Examination, pH, Viscosity and In Vitro Release

RP-SLN and RP-NLC enriched hydrogel formulations were inspected for their homogeneity, consistency and presence of lumps by visual inspection after the gels have been set in the container. The pH of the prepared hydrogel formulation was determined using a digital pH meter (PH540GLP, WTW, Weilheim, Germany) in triplicate at 25 °C. About 1 g of hydrogel formulation was dispersed in 10 mL of distilled water and the pH was measured by dipping the glass electrode completely into the gel system to cover the electrode. The viscosity of hydrogel formulations of RP-SLN and RP-NLC were determined at 25 °C, using a Brooke field viscometer DVII model with a T-Bar spindle of T-64 in combination with a helipath stand. For the measurement, 1 g of gel was dispersed in 10 mL of water in a glass beaker. The helipath T-bar spindle was moved up and down, giving viscosities at the number of points along the path [36].

#### 2.7.2. Spreadability

Generally, time in seconds taken by both glass slides to slip-up off from hydrogel formulation placed in between the slides under the direction of a certain load is considered as the spreadability of the formulation. Accurately weighed 1 g of prepared hydrogel formulation was placed between two glass slides and known weight placed over an upper glass slide and left for about 5 min [37]. The time required by the upper slide to move on the application of weight to it through the pulley was noted, and spreadability was calculated by using the following Equation (3), in triplicate:

S = W × L/T(3)

where, S—spreadability; W—weight applied to upper slide; L—length of the glass slide and T—time taken to separate the slides from each other.

#### 2.7.3. Extrudability Test

Extrudability test used to determine the force essential to extrude the gel from the packed tube container. For this purpose, a known amount of gel formulation was filled in a standard capped collapsible aluminum tube and sealed by crimping both ends. The weight of the tube was recorded. Then the tubes were placed between the two glass slides were clamped. Then, 500 g of weight was placed over the glass slides, and the cap was removed. The amount of gel extruded was collected and weighed [38]. The extrudability of each formulation was measured, in triplicate, and calculated by using the Equation (4):

E = W/A(4)

where, E—extrudability, W—applied weight to extrude gel from the tube and A—area.

#### 2.7.4. Total Drug Content

The RP content in the RP-SLN-C and RP-NLC-C formulation was determined by dissolving (accurately weighed) 0.5 g of the gel formulation in 15 mL of ethanol and vortex for 5 min at 5000 rpm, sonicated in a bath sonicator (Sonica, 3200MH, Singen, Germany) for 15 min, to get complete solubility of the drug. The analyte was prepared and estimated for drug content, as per the drug content procedure of lipid nano formulations.

#### 2.7.5. Lyophilization of RP-SLN and RP-NLC Formulation

RP-SLN and RP-NLC formulation was subjected to freeze-drying. Trehalose dihydrate used as a cryoprotectant (10% *w/w*) and added to the aqueous phase of the nanoformulations. Prepared formulations were kept in an ultra-freezer at −80 °C (Sanyo, Japan) overnight. The frozen samples were then transferred into the freeze-dryer (Lyodel, Delvac Pumps Pvt. Ltd., Chennai, India). The vacuum was applied, and the sample was subjected to various drying phases for about 36 h, to get powdered lyophilized RP-SLN and RP-NLC product [39]. The powdered formulation was reconstituted and used for further experimental studies.

#### 2.7.6. In Vitro Release Studies

In vitro release studies of RP-SLN and RP-NLC, P-SLN-C and RP-NLC-C, comparison with RP-S and RP-C, respectively, were performed using the dialysis method with Franz diffusion cells. A dialysis membrane (Hi-Media, Mumbai, India) having a pore size of 2.4 nm, diameter of 72 dm and molecular weight cut-off between 12,000–14,000 kDa, was used for the release studies [10]. The dialysis membrane was soaked overnight in double distilled water prior to the release studies. Phosphate buffer (PB) pH 6.8 was used as release media [40]. The experimental unit consists of a donor and receptor compartment. The donor compartment filled with respective formulation (0.5 mL and 0.5 g for nanoparticles and hydrogel, respectively), and the receptor compartment consists of a 12 mL of release medium. The diffusion cell temperature was maintained at 37 ± 0.5 °C. At fixed time points, 1 mL of sample was withdrawn from the receiver compartment and replenished with the same volume of release medium. The collected samples were analyzed for RP content using HPLC. Drug release vs. time profiles of RP from lipid nanoparticles and hydrogel formulation was plotted and subjected to release kinetic mathematical models, to determine the release mechanism of RP.

### 2.8. Ex Vivo Permeation Studies

#### 2.8.1. Preparation of Skin

Male albino Wistar rats weighing between 210 ± 30 g were used for the skin collection. Rats warehoused with free access to food and water and sacrificed by anesthesia using ether. The rat abdominal surface hairs were removed by a depilatory. The skin was surgically removed. The subcutaneous tissue was removed manually, and the dermis side was wiped with isopropyl alcohol for removing residual adhering fat. Full-thickness of skin was washed with PBS, wrapped in aluminum foil and stored at −20 °C until further use.

#### 2.8.2. Procedure

The ex vivo permeation studies of RP-SLN and RP-NLC, P-SLN-C and RP-NLC-C, comparison with RP-C formulation was studied using Franz-diffusion cells, with a diffusional area of 3.56 cm^2^. The rat skin was fixed between the donor and receptor chambers of the diffusion cell. The rat skin was adjusted with the stratum corneum layer facing the donor compartment and the dermis faced the receptor compartment [41]. The rest of the study was conducted as per the above described in vitro release study protocol. The results of RP permeation were kinetically treated to determine the order of mechanism of permeation. The flux was calculated from the slope of the linear part of the cumulative amount of RP permeated per unit area (µg/cm^2^), against a time (h) plot. Permeation rate was calculated to form the slope of percent drug permeated against time. The statistical significance of the formulations was compared with unpaired student t-test at *p* < 0.05 level.

### 2.9. Stability Studies

The physical stability of RP-SLN, RP-NLC and the corresponding gel formulations were evaluated by storing the samples at refrigerated and room temperature over three months. In this study, RP-SLN and RP-NLC formulation was filled in scintillation glass vails, RP-SLN-C and RP-NLC-C formulations were packed in aluminum collapsible tubes. All the samples were stored at 25 ± 2 °C and 4 ± 2 °C over three months. Samples were withdrawn at predetermined time intervals and analyzed [42]. Parameters such as particle size, PDI, ZP, assay, DL, EE and drug content, pH, viscosity, spreadability, extrudability for lipid nanoparticles and hydrogel formulations, respectively, were observed [28]. The data were compared for a statistical significance at p value of less than 0.05.

### 2.10. Solid-State Characterization

#### 2.10.1. Differential Scanning Calorimetry (DSC)

RP, pure solid lipid, physical mixture of drug and lipid (1:1) and lyophilized RP-SLN and RP-formulation were subjected to a DSC (DSC 822 e/200, Mettler, Switzerland) analysis. The instrument was calibrated with indium (calibration standard, purity > 99.99%) before the analysis. About 8–10 mg of sample was taken for analysis into standard aluminum pans. An empty pan was used as a reference. The heating rate was increased at the rate of 10 °C/min and the obtained thermograms were observed for crystallinity changes and compatibility of RP with other excipients.

#### 2.10.2. X-ray Diffraction (XRD)

XRD technique was used to determine the crystalline behavior of RP after loading into nano formulation. XRD analysis of samples was conducted by using a powder X-ray diffractometer (XRD 6000, Shimadzu, Tokyo, Japan). In this technique, the samples were exposed to nickel filtered CuKα radiation (40 kV, 30 mA) and scanned from 2° to 50°, 2*θ*, at a step size of 0.045° and step time of 0.5 s. Samples used for XRD analysis were RP, pure solid lipid, physical mixture of lipid and RP, and lyophilized nano formulations.

#### 2.10.3. Morphology of Lipid Nanoformulations and Hydrogels by Scanning Electron Microscopy (SEM)

The morphology of RP-SLN and RP-NLC and their corresponding hydrogel formulation was studied by SEM (S4800, Hitachi, Japan; SEI, Hitachi, Tokyo, Japan). Initially, the nano formulation was fixed on to the carbon coated brass stub. This was sputter coated with Platinum coating machine (JEOL, JFC-1600 Auto fine coater) and mounted in SEM (JSM- 6510LA, JEOL, Tokyo, Japan), for surface analysis by applying at different voltages [43].

### 2.11. In Vivo Studies

#### 2.11.1. Pharmacokinetic (PK) Studies

##### Study Protocol

PK studies of RP-SLN and RP-NLC comparison with RP-S orally, RP-SLN-C and RP-NLC-C comparison with RP-C formulations transdermally were performed in male Wistar rats, after a single dose administration. Prior to the study, written approval is confirmed, as per the guidelines of CPCSEA, Synapse Life Science, Warangal, India. Male Wistar rats weighing 210 ± 30 g were used for the study. Rats were procured and kept under standard laboratory condition (25 °C/55%RH), housed in polypropylene cages with free access to a standard laboratory diet and water ad libitum.

##### Study Design

Rats were visually examined for skin irregularities and eliminated the rat with skin lesions or acne conditions before the initiation of the study. About 10 ± 1 cm^2^ of rat skin was shaved on the dorsal side and kept for over-night fasting and was used for transdermal application. The rats were divided into six groups, each with six animals. Rats were treated with the following treatment: group I, II and III received RP-SLN, RP-NLC and RP-S orally (1.1 mg/kg), respectively; group IV, V and V received RP-SLN-C, RP-NLC-C and RP-C topically (1.1 mg/kg), respectively [44]. For oral administration, a sterilized oral feeding gauge was used. Blood samples (0.4 mL) were collected at predetermined time points (0, 0.5, 1, 2, 3, 4, 6, 8, 10, 12 and 24 h) through the marginal ear vein after ether anesthesia. The serum was separated by centrifugation (Remi equipment, India) for 10 min at 10,000 rpm. The serum was stored at −20 °C until analysis.

##### Extraction and Quantification of RP from Rat Serum

RP content from rat serum was analyzed by using HPLC method, as per the Luzardo-Alvarez et al. reported method [45]. A Shimadzu model HPLC equipped with quaternary LC-10A VP pumps, variable wavelength programmable UV/VIS detector SPD-10AVP column oven (Shimadzu), SCL 10AVP system controller (Shimadzu), Rheodyne injector fitted with a 20 µL loop and spinchrome software were used.

The RP was extracted from the serum by using tert-butyl-methyl-ether as precipitating agent. Briefly, 100 µL of the serum and 100 µL of paracetamol as internal standard (IS) was added and mixed for 3 min. To this, then 500 µL of tert-butyl-methyl-ether was added and stirred for 5 min and then centrifuged at 5000 rpm for 15 min at 25 °C. The supernatant organic layer was removed. This process was repeated twice. Finally, the organic phase was dried, and the residue obtained was dissolved in the 100 µL of mobile phase. Analysis was performed on a phenomenex C_18_ column (5 µm, 250 mm × 4.6 mm i.d.). The mobile phase consisting of acetonitrile:0.05 M ammonium acetate buffer pH 2.5 (25:75, *v/v*) was pumped through the column, at a flow rate of 1 mL/min. Aliquots of 20 µL from each sample were injected into the system via the manual injector. All the samples were filtered through a 0.22 µ membrane filter prior to injection. Detection was performed at 254 nm. The retention times of RP and IS were 3.8 and 7.0 min, respectively. At these retention times, there was no interference of any substance in the serum. The limit of quantification and detection was 100 ng/mL and 50 ng/mL, respectively. The linearity was obtained in the concentration range of 0.5–5 μg/mL and the *r*^2^ value of the standard graph was >0.997.

##### Calculation of PK Parameters

The PK parameters of RP from lipid nanoparticles, hydrogels and control formulations after oral and topical administration such as peak serum concentration (*C*_max_), time for peak serum concentration (*t*_max_), *AUC*_total_, elimination half-life (*t*_½_) and mean residence time (MRT) were calculated by Kinetica software (version 5.0, USA). The values were expressed as mean ± SD.

#### 2.11.2. Skin Irritation Studies

Draize patch test was used to estimate the skin irritation of RP-SLN-C, RP-NLC-C, comparison with RP-C formulation as control formulation, aqueous formalin solutions (0.8% *w/v*) as positive control and untreated group as negative control. The study was conducted in male Wistar rats [46]. Animals were divided into five groups, each with three animals and 210 ± 30 g weight. The hair on the dorsal side of rats was removed by clipping 24 h before the experiment and observed for skin abnormalities. The rats were applied with the formulations with 5 ± 1 cm^2^ area, with the following treatment: group I kept as negative control, group II received RP-C formulation (1.1 mg/kg), group III received RP-SLN-C formulation, group IV received RP-NLC-C formulation and group V received formalin solution (0.8% *w/v*). Change in skin color, morphology and changes in the signs of erythema and edema were observed for 48 h. The application sites were graded according to a visual scoring scale against the RP-C control formulation.

##### Statistical Treatment of Data

The statistical significance comparison of data was made with unpaired student *t*-test using Graph pad prism software (version 5. 02.2013, USA). A *p*-value of less than 0.05 was considered statistically significant.

#### 2.11.3. Pharmacodynamic Study

Pharmacodynamic (PCD) studies of RP loaded lipid nanoparticles, corresponding hydrogel and control formulations were carried out in haloperidol induced PD Wistar rats. Haloperdiol 1 mg/kg was given intraperitoneally to induce PD [47]. Rats were divided into eight groups, each with six numbers. The treatment of the study as follows: group I served as normal control, group II as PD control, group III received RP-SLN formulation, group IV received RP-NLC formulation, group V received RP-SLN-C, group VI received RP-NLC-C, group VII received RP-C formulation and group VIII received RP-S formulation. After 1 h of treatment, 1 mg/kg haloperidol was administered to induce PD.

#### 2.11.4. Biochemical Studies

##### Tissue Preparation

Once the PD was induced, the rats were sacrificed, and their brains were taken out for harvesting striatum and substantia nigra by cutting acoronal section of 1 mm thickness, using rat brain matrix in the light of rat brain atlas. For enzymatic assays, striatum was homogenized (10%, *w/v*) in 0.01 M phosphate buffer (pH 7.0) and centrifuged at 11,000 rpm for 20 min at 4 °C to get post-mitochondrial supernatant (PMS). This was used for the estimation of dopamine levels, TBARS (thiobarbituric acid reactive substances), GSH (glutathione, antioxidant enzyme) and catalase activity.

##### Lipid Peroxidation

Lipid peroxide levels of the RP from various formulations were estimated, as per the previously reported method [48]. Briefly, 200 µL of PMS was pipetted in an eppendorf tube and incubated at 37 ± 1 °C in a water bath shaker for 60 min at 100 rpm; another 200 µL was pipetted in an eppendorf tube and placed at 0 °C incubation. After 1 h of incubation, 400 µL of 5% trichloroacetic acid and 400 µL of 0.67% TBA was added in both samples (i.e., 0 °C and 37 °C). The reaction mixture from the vial was transferred to the tube and centrifuged at 1200 rpm for 15 min. The supernatant was transferred to another tube and placed in a boiling water bath for 10 min. Thereafter, the test tubes were cooled, and the absorbance of the color was measured at 535 nm. The rate of lipid peroxidation was expressed as nmol of TBARS reactive substance formed/h mg protein.

##### Assay for Reduced Glutathione Content (GSH)

PMS (200 µL) was precipitated with 200 µL of sulfosalicylic acid (4%). The samples were kept at 4 °C for at least 1 h and then subjected to centrifugation at 1200 rpm for 15 min at 4 °C. The assay mixture contained 100 µL of filtered aliquot (10%, *w/v*), 1.7 mL phosphate buffer (0.1 M, pH 7.4) and 200 µL DTNB (DTNB) (4 mg/ mL of phosphate buffer, 0.1 M, pH 7.4), in a total volume of 2 mL. The yellow color developed was ready immediately at 412 nm [29].

##### Determination of Catalase Activity

The Coliborne 1985 reported method was used to estimate the catalase activity [49]. Briefly, the assay mixture consisted of 200 µL phosphate buffer (0.1 M, pH 7.4), 950 µL hydrogen peroxide (0.019 M) and 50 µL of PMS, in a total volume of 3.0 mL. Changes in absorbance were recorded at 240 nm. Catalase activity was calculated in terms of nmol H_2_O_2_ consumed/(min mg protein).

##### Dopamine Levels

To the 200 µL of PMS, 50 µL 0.4M HCl and 100 µL of EDTA/sodium acetate buffer pH 6.0 were added, followed by 100 µL iodine solution (0.1M in ethanol) for oxidation. The reaction was stopped after 2 min by the addition of 100 µL of Na_2_So_3_ (sodium sulphite) solution. To this, 100 µL acetic acid was added after 1.5 min. The solution was then heated to 100 °C for 6 min. When the sample again reached room temperature, excitation and emission spectra were read from the fluorimetry. The readings were taken as primary filter as 360 nm, a secondary filter 440 nm for dopamine.

## 3. Results and Discussion

The main intent of the current investigation was to design, develop and optimize the oral delivery of RP using lipid nanocarrier systems. Further, to evaluate the alternative route for the enhanced activity for the PD treatment. RP has poor oral bioavailability due to hepatic first-pass metabolism. Hence, colloidal carrier systems might be bypassing the hepatic metabolism of the poorly bioavailable drugs. In this attempt, SLN and NLC vehicles and their corresponding hydrogel systems were developed.

Initially, solid lipid, liquid lipid and surfactants were selected based on the earlier reported studies, as well as screening studies. Dynasan-114, Caproyl 90, soylecithin and poloxamer 188 was selected as solid lipid, liquid lipid, primary and secondary surfactant, respectively, for the preparation of RP-SLN and RP-NLC formulation. Process parameters, such as homogenization time and speed and probe sonication time and amplitude, were optimized based on physical characteristics and stability criteria. Homogenization alone did not reduce the particle size for the ideal transdermal application. Homogenization coupled with probe sonication yielded the nanoparticles with desired transdermal application range (data not shown). Homogenization time and speed at 12,000 rpm for 10 min and probe sonication for 15 min at 40% amplitude were selected as optimized process conditions for the preparation of RP-SLN and RP-NLC formulations. RP-SLN composed of 2.0% *w/v* of Dynasan-114 as solid lipid and RP-NLC composed of 1.25% *w/v* of Dyanasn-114 and 0.75% *w/v* of Caproyl 90 as liquid lipid, respectively. In general, solid lipid to liquid lipid ratio 3:1 is ideal for the preparation of NLC formulation [31].

### 3.1. Characterization of RP-SLN and RP-NLC

#### 3.1.1. Size, PDI and ZP

The composition of optimized RP-SLN and RP-NLC formulation is shown in Table 1. Both of the formulations were prepared and evaluated for physical characteristics. Particle size, PDI and ZP was measured using Zetasizer with 50 times dilution of formulation. The results are presented in Table 2. RP-SLN and RP-NLC showed particle size, PDI, ZP of 210.6 ± 3.6 nm, 0.21 ± 0.06, −28.4 ± 0.9 mV and 193.2 ± 2.1 nm, 0.18 ± 0.03 and −30.5 ± 1.5 mV, respectively. Particle size of the lipid nanocarrier formulations was below 250 nm. This particle size is ideal for the application of the topical route [38]. Further, the particle size of NLC formulation was lower, but less non-significant than the SLN formulation. This could be due to the presence of liquid lipid; more drugs would be accommodated in the void spaces of the NLC formulation [50]. The PDI of both formulations was below 0.5. This could be considered as homogeneous distribution of the formulation [16]. ZP of the lipid nanoparticle dispersions might be in the range of ± 30 mV and was considered stable. In case of RP-SLN and RP-NLC formulations, ZP was observed to be −30 mV. Poloxamer 188, a non-ionic surfactant, was present, which promotes the steric stabilization, because it can reduce the electrostatic repulsion between the particles by establishing a coat around the surface for keeping the stability of lipid nanoparticles [20].

#### 3.1.2. Drug Content, DL and EE

Drug content, DL and EE of the RP-SLN and RP-NLC formulations shown in Table 2. Drug content and DL of the RP-SLN and RP-NLC were found to be 97.5 ± 1.3 and 98.3 ± 2.0%, 7.7 ± 0.5 and 8.4 ± 0.3%, respectively. EE of the RP-SLN was found to be (76.8 ± 2.7%) less than RP-NLC (84.1 ± 2.3%). This might be due to the fact that combination of solid lipid and liquid lipid, resulting in the formation of weaker crystallization. This, in turn, results in the accommodation of more drug than SLN. Further, the imperfect lipid matrix structure of the NLC formulation, presenting a gap between the triglyceride fatty acid chains in crystal and thus, increasing the ability of the drugs for entering the matrix.

#### 3.1.3. Characterization of RP-SLN-C and RP-NLC-C

RP-SLN and RP-NLC formulation converted to RP-SLN-C and RP-NLC-C formulation, using 1% *w/v* carbopol 934 as the gel-forming polymer. The final concentration of RP was maintained at 0.2% *w/v*. The prepared gel formulations were neutralized by triethanolamine (0.3% *w/v*). Triethanolamine is a non-ionic neutralizer and it does not disrupt the ionic balance, and no aggregation of the nanoparticles observed. Therefore, ionic neutralizers are avoided in the present investigation [51].

#### 3.1.4. Visual, Rheological and pH Studies

RP loaded lipid nanoparticle hydrogel formulation were kept aside for 24 h and observed for color, odor and consistency. They did not show any signs of sedimentation after centrifugation either. The hydrogel formulations evaluated for pH, viscosity, spreadability, extrudability and drug content and results directed in Table 3. The pH of the formulation was measured by a digital pH meter. The pH of the formulations was neutralized by the addition of triethanolamine. The pH of RP-SLN-C and RP-NLC-C formulation was found to be 6.6 ± 0.3 and 6.5 ± 0.4, respectively. This is the ideal pH for dermal applications [50]. The viscosity of the formulations was found to be 2678 ± 64.6 cP and 2352 ± 77.1 cP for RP-SLN-C and RP-NLC-C, respectively. The spreadability of the RP-SLN-C and RP-NLC-C formulations was 6.3 ± 0.5 g cm/sec and 6.1 ± 0.2 g cm/sec, respectively. This indicates the easy application of hydrogel formulations, with the use of slight shear on the skin surface. Extrudabilities were found to be 1.4 ± 0.3 and 1.2 ± 0.2 g/cm^2^ RP-SLN-C and RP-NLC-C formulation, respectively, in 10 s, on applying a weight of 500 g. A similar observation was reported in earlier studies [36]. In general, the more the gel is extruded, the better the extrudability was; hereafter, a lower amount of energy was needed to extrude the gel without difficulty from the filled container. The drug content of the RP was 98.2 ± 2.1 and 99.6 ± 2.4% from RP-SLN-C and RP-NLC-C, respectively.

### 3.2. In Vitro Release Studies

**Figure 1:**
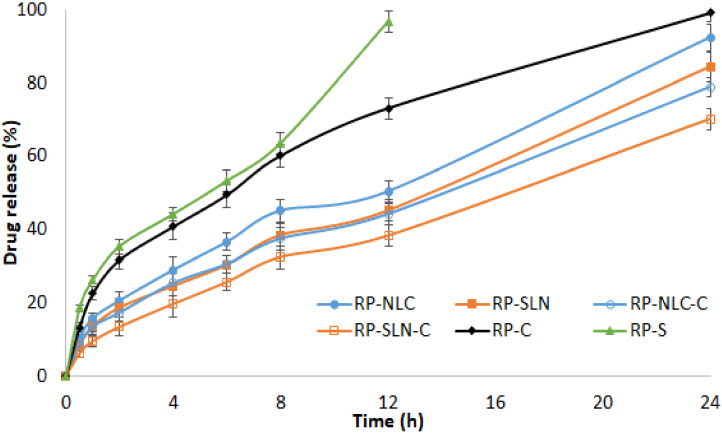
In vitro release profiles of RP from RP loaded lipid nanoparticles, hydrogel formulations and control formulations through the dialysis membrane (mean ± SD, *n* = 3).

Franz diffusion cells were used to study the in vitro release profiles of RP from lipid nanoparticles and corresponding hydrogel formulations, through dialysis membrane. RP-C and RP-S formulations were used as control formulations and PB pH 6.8 as a release medium. The release profiles of formulations are shown in Figure 1. Form the results, the cumulative percent of RP release from RP-SLN and RP-NLC formulations were 84.3 ± 3.2% and 92.3 ± 2.6%, respectively, over 24 h. In the initial 2 h, the drug release was less than 20% from SLN formulation and increased with time after 2 h. This could probably be due to the slow diffusion of the drug from the lipid matrix. The RP-NLC formulation was released to be significantly (*p* < 0.05) higher than the RP-SLN formulation. This is because of the lipid soft shell and its capability of promoting high solubility of lipophilic drugs character [23,24], in which a high amount of the drug is easily loaded and released by diffusion or matrix erosion. Furthermore, the incorporation of liquid lipid into the solid lipid matrix caused the RP-NLC to become more imperfect and allowed loaded drugs to become easier to release, thus increasing the drug release rate when liquid lipid was included in the NLC matrix. For the above reasons, it achieved the results of sustained release and increased drug release rate compared with RP-SLN. Further, RP-S formulation showed 96.7 ± 2.8% over 12 h. This indicates the sustained and prolonged release of the RP from the lipid nanoparticles.

The percentage of RP release from the RP-SLN-C and RP-NLC-C hydrogel formulation was 70.1 ± 2.9% and 78.8 ± 2.5% during 24 h, respectively. Comparing the drug release from the RP-C formulation, it showed 99.1 ± 2.3% over 24 h and indicates sustained release from nanoparticle enriched hydrogel formulation. This could be due to the drug dispersed in the lipid matrix diffusing from the gel formulation more slowly than drug suspending in Tween^®^ 20 dispersion. RP-C formulation sustains the drug release compared with RP-S formulation, due to the presence of the gelling agent. The drug release from the hydrogel formulations was relatively slow compared with nanoparticle formulations. This might be due to the fact that the RP molecules were entrapped in the lipid matrix, which was in turn incorporated into the hydrogel system.

The release profiles were subjected to kinetics mathematical modelling to determine the release mechanism and diffusional release exponent. The data fit into to zero-order, first-order, Higuchi and Korsemeyer-Peppas model. The regression coefficient values are shown in Table 4. The high r^2^ values were observed with a Korsemeyer-Peppas above 0.98, with n values above 0.5. This indicated that the release of the drug from lipid nanoparticles as well as nanoparticle enriched hydrogel formulations followed a non-Fickian with an anomalous diffusion mechanism.

### 3.3. Ex Vivo Permeation Studies

**Figure 2:**
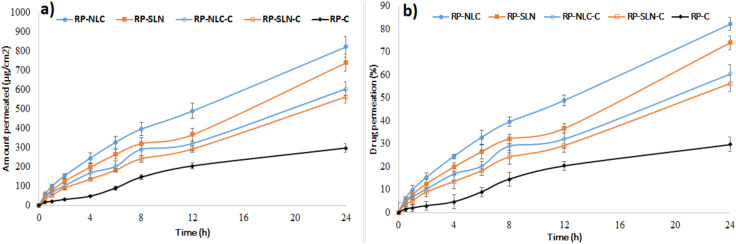
Amount of drug permeate (**a**) and percentage drug permeation (**b**) of RP from RP loaded lipid nanoparticles, hydrogel formulations and control formulations through rat skin (mean ± SD, *n* = 3).

Permeation studies of RP lipid nanoparticles, hydrogel and control formulations were performed through rat skin using Franz diffusion cells. Figure 2a,b displayed the amount of drug permeated vs. time, and the percentage of drug permeated vs. time profile of RP from various formulations. The amount of RP permeate, percentage RP permeation, flux and permeability data of formulations are shown in Table 5. The amount of RP permeated from RP-SLN, RP-NLC, RP-SLN-C and RP-NLC-C formulations were 739.2 ± 34.1 µg/cm^2^, 820.7 ± 57.2 µg/cm^2^, 561.5 ± 28.9 µg/cm^2^ and 603.2 ± 41.7 µg/cm^2^, respectively over 24 h. RP-C control formulation showed 296.2 ± 23.5 amount of drug permeated, respectively over 24 h. A significantly (*p* < 0.05) higher amount RP permeated was observed with lipid nanoparticle and hydrogel formulations than control gel formulation. This indicates the sustained release behavior of the lipid nanoparticles and hydrogel formulations. Furthermore, the amount of drug permeate from lipid nanoparticle enriched hydrogel formulations was lower than the lipid nanoparticle formulation. This could be due to the time required for the drug to diffuse slowly from the gel. Compared with SLN and SLN-C, NLC and NLC-C permeation was higher, due to the presence of liquid lipid, that facilitates the easy permeation of drug through the skin.

The flux of the permeation of the formulations was calculated by plotting the graph of the amount drug permeate per sq.cm against time, and the slope of the straight line gives the flux (Figure 2a). The flux of the formulations is shown in Table 5. The flux of the RP-SLN and RP-NLC formulation was 2.2 and 2.5-folds compared with RP-C formulation, respectively. An improvement of 1.7 and 1.8-folds from RP-SLN-C an RP-NLC-C was compared with RP-C formulation, respectively. Moreover, the flux of RP-NLC and RP-NLC-C was 1.1 and 1.0-folds higher than RP-SLN and RP-SLN-C formulations, respectively.

Percentage drug permeated from RP-SLN, RP-NLC, RP-SLN-C and RP-NLC-C formulation were 73.9 ± 3.1, 82.0 ± 2.9, 56.1 ± 3.6 and 60.3 ± 4.2%, respectively over 24 h, while the percentage of permeability was higher than the control formulations (29.6 ± 3.2%). The permeability or permeation rate of the formulations was estimated by the percentage of drug permeate against time. The slope of the linear portion considered as permeation rate and values is shown in Table 5. Permeability of the RP-SLN, RP-NLC, RP-SLN-C and RP-NLC-C formulation was significantly higher than the RP-C formulation. From the mathematical data fit, drug release from lipid nanoparticles and hydrogel formulations showed the Korsemeyer-Peppas model with anomalous diffusion pattern (Table 4).

The permeation of the drug from the control formulation is significantly less when compared with hydrogel formulation. This could be due to the following reasons: better penetrability of lipid matrices through skin and thus maintained therapeutic concentrations in the upper layers of the skin, greater skin retention of drug in the case of nanoparticle enriched incorporated hydrogels, occlusive and bioadhesive properties of nanoparticles, where occlusion decreases the transepidermal water loss, which further increases skin hydration and permeability. Further, it could also lead to the accumulation of drug into the upper skin layers, reducing drug flux and creating a reservoir to prolong skin residence time.

### 3.4. Stability Studies

The stability of RP lipid nanoparticles and corresponding hydrogel formulations were established by the store the samples at refrigerator and room temperature over three months. The RP-SLN and RP-NLC formulation was withdrawn and analyzed for physicochemical characteristics and shown in Table 6. RP-SLN and RP-NLC formulations showed no significant changes in the physical and chemical parameters of the formulation over three months’ observation. Relatively, small changes (2–3%) were noticed at room temperature compared with the refrigerator temperature.

Similarly, RP-SLN-C and RP-NLC-C formulations were analyzed for pH, viscosity, spreadability, extrudability and drug content. The formulations were found to be stable for three months at both storage conditions. The data are exemplified in Table 7.

### 3.5. Lyophilization of KZ-SLN Formulation

The lyophilization process is useful for the conversion of the lipid nanoparticle dispersion into powder form and there is a chance to either fill into capsules or compressed to tablets and used for the solid-state characterization and morphology studies. RP-SLN and RP-NLC formulation produced a clear floppy cake without showing any sticking or moisture content formation. The lyophilized formulation was reconstituted as equal to the same concentration of RP in lipid nanoparticle dispersion and analyzed for physical and chemical characteristics. Particle size and PDI of the reconstituted formulation was increased double-fold compared with the pre-lyophilized formulation. However, no significant changes were observed in the ZP, drug content and EE of the lyophilized formulation (Table 8). The changes in the particle size and PDI might be due to the aggregation of the particles by the process of lyophilization. These results were also noticed in earlier studies [52,53].

### 3.6. Solid-State Characterization

#### 3.6.1. DSC

**Figure 3:**
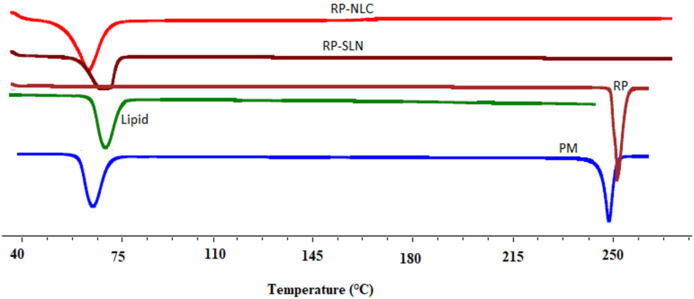
DSC thermogram of pure drug (RP), pure lipid (Lipid), physical mixture of drug and lipid (PM), lyophilized optimized RP-SLN formulation (RP-SLN) and RP-NLC formulation (RP-NLC).

DSC technique was used to determine the crystallinity and compatibility of RP with other formulation excipients. DSC results of pure drug, pure lipid, physical mixture of drug and lipid, lyophilized RP-SLN and RP-NLC formulation presented in Figure 3. RP showed a sharp endothermic peak of RP at 244.6 °C and which is corresponding to the melting temperature range of RP (243–255 °C) [29].

Similarly, the endothermic peak of the physical mixture showed a drug melting peak at 243.5 °C. This slight shift in the temperature might be due to the mixing of the drug with lipid. However, lipid showed a similar melting temperature peak at 58.6 °C, like the pure lipid endothermic peak. The RP peak was absent in both lyophilized formulations. This indicates the conversion of the drug into amorphous form in SLN and NLC formulation.

#### 3.6.2. XRD

**Figure 4:**
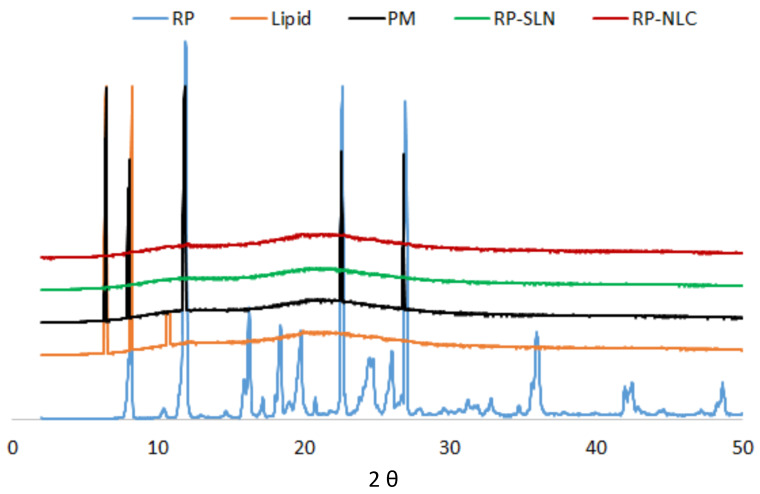
XRD spectra of pure drug, pure lipid, physical mixture of drug and lipid, lyophilized optimized RP-SLN formulation and RP-NLC formulation.

The XRD results of pure RP, pure lipid, physical mixture of drug and lipid, and lyophilized RP-SLN and RP-NLC formulation are shown in Figure 4. XRD spectra of pure RP showed characteristic peaks at 2*Ɵ* values of 7.41, 11.56, 22.49, 23.73, 24.85, and 26.88 confirms and indicated the crystalline nature of RP [54]. The unique peaks of RP were absent in the lyophilized sample and present with less intensity in the physical mixture. This suggested that the drug was not in crystalline form after the lyophilization of lipid nanoformulations. The intensity of pure lipid peaks was also decreased in the lyophilized samples. This reduced intensity indicated the decreased crystallinity of lipid. The change in the crystallinity of lipid and drug would influence the release of RP from nanoparticles. This reduction in crystallinity was noticed in DSC analysis also.

#### 3.6.3. SEM

**Figure 5:**
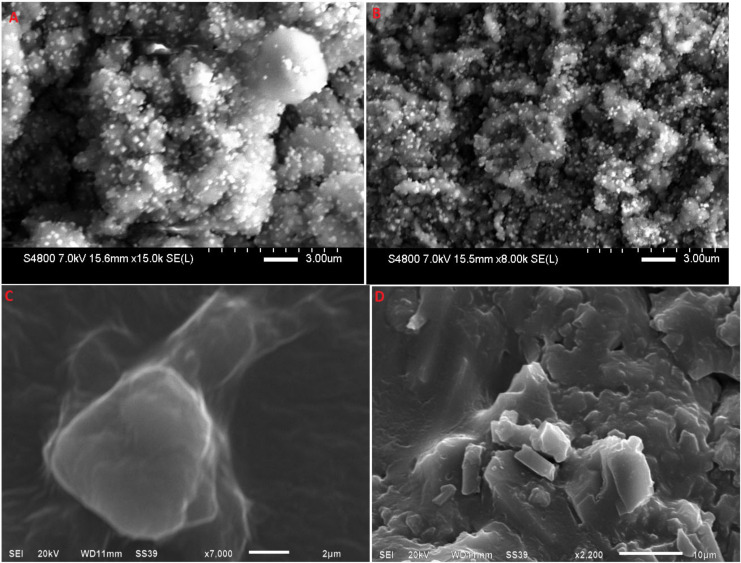
SEM images of (**A**) lyophilized RP-SLN formulation, (**B**) lyophilized RP-NLC formulation, (**C**) RP-SLN-C formulation, and (**D**) RP-NLC-C formulation.

SEM images of lyophilized RP-SLN and RP-NLC and corresponding hydrogel formulations were shown in Figure 5. Hydrogel formulation showed an uneven surface with higher scuffle boundaries (Figure 5C,D), while lipid nano formulations demonstrated a virtually sphere-shaped surface with aggregates along the particle surface (Figure 5A,B). The SEM images reveal that the drug-filled nanoparticles were entrapped in the carbopol hydrogel matrices, which is additional evidence from the sustained release profile of RP lipid nano formulations.

### 3.7. In Vivo Studies

#### 3.7.1. Skin Irritation Studies

Skin irritation studies of RP-SLN-C and RP-NLC-C formulation was carried out in Wistar rats, using RP-C as control formulation, 0.8% *w/v* formalin as positive control and the untreated group as a negative control. The results of the erythema and edema scores of the study were observed for 48 h and shown in Table 9. Lipid nanoparticle enriched hydrogel formulations did not cause any changes in the skin color and morphology during the entire duration of observation. However, RP-C control formulation exhibited signs of erythema and edema after 24 h application and 48 h application, respectively. Therefore, the results indicated that RP-SLN-C and RP-NLC-C formulation had acceptable skin tolerability and patient acceptance.

#### 3.7.2. PK Studies

**Figure 6:**
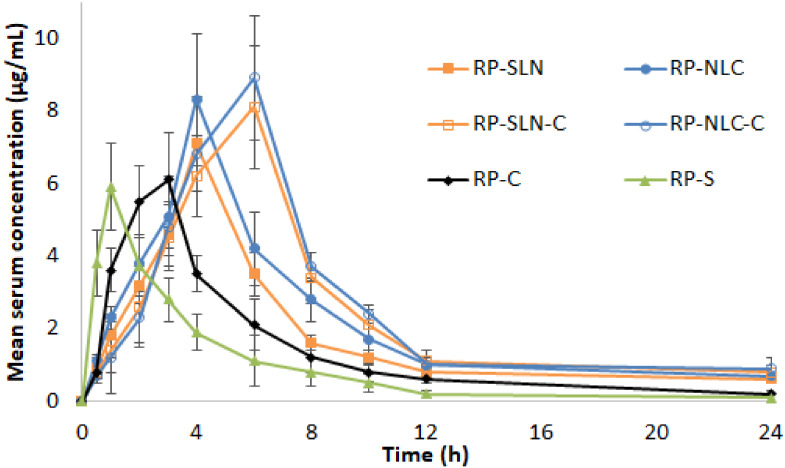
Mean serum and time profiles of RP from RP-SLN, RP-NLC, RP-S and RP-SLN-C, RP-NLC-C, RP-C formulations after oral and topical administration, respectively in Wistar rats (mean ± SD, *n* = 6).

PK studies of RP-SLN and RP-NLC in comparison with RP-S orally; RP-SLN-C and RP-NLC-C in comparison with RP-C transdermally were performed in Wistar rats. The rats were administrated with a single dose at 1.1 mg/kg body weight. Serum samples were collected and analyzed for RP connect using the HPLC method. Paracetamol was used as IS for the extraction process. Mean serum concentration vs. time profiles of RP after single dose topical and oral administration of formulations were portrayed in Figure 6 and calculated PK parameters showed in Table 10. From the results, *C*_max_ of RP-SLN and RP-NLC formulation was 7.1 ± 0.9 µg/mL and 8.3 ± 0.7, respectively. It is significantly (*p* < 0.05) higher from RP-S (5.9 ± 1.2 µg/mL) formulation. However, the *C*_max_ of RP-SLN-C (8.1 ± 1.7 µg/mL) and RP-NLC-C (8.9 ± 1.4 µg/mL) formulation was almost similar to the RP-SLN and RP-NLC formulation. However, compared with RP-C formulation, a significant (*p* < 0.05) level were achieved. *T*_max_ of the lipid nanoparticles and respective hydrogel formulations were 4 h and 6 h, respectively. Compared with control RP-C formulation, 2-fold and 1.3-folds enhancement from hydrogel and lipid nanoparticle formulations was observed. Elimination half-life and MRT of the testing formulations was 1-5-folds higher than the control formulations and significantly higher (*p* < 0.05). This indicates the sustained release of the drug from SLN and NLC, SLN and NLC enriched hydrogel formulations. AUC is of the formulation is indicative of the bioavailability of the formulation. *AUC*_tot_ of the RP-SLN and RP-NLC formulation was found to be 49.8 ± 5.8 a µg/mL·h and 62.7 ± 4.2 µg/mL·h, respectively. From this is revealed that 2.1 and 2.7-folds enhancement in the oral bioavailability from RP-SLN and RP-NLC formulations, respectively than RP-S control formulation (22.8 ± 2.4 µg/mL·h). Similarly, 69.8 ± 5.6 µg/mL.h and 76.8 ± 4.8 µg/mL.h of *AUC*_tot_ noticed from RP-SLN-C and RP-NLC-C formulation, respectively. It is higher (*p* < 0.05) than RP-C control formulation (34.8 ± 2.9 µg/mL·h). From this, about 2.0 and 2.2-folds improvement in the transdermal bioavailability from RP-SLN-C and RP-NLC-C formulation was observed than RP-C control formulation. Further, RP-SLN-C and RP-NLC-C showed 1.8 and 1.2-folds improvement in the transdermal bioavailability than RP-SLN and RP-NLC formulation oral bioavailability, respectively. This is also evidenced by in vitro and ex vivo permeation study results. SLN and NLC formulation also favors the enhanced oral delivery of the RP than the conventional dosage form. This might be due to the small particle size, the presence of surfactant enhanced the effective surface area, lipid promotes gastric motility and also avoids the first-pass metabolism by increased lymphatic uptake [22,55,56]. The mechanism for enhanced dermal delivery due to the fact that the presence of carbopol leads to the formation of the special network and from which the drug is slowly diffused subsequently sustains drug release; the further mucoadhesive nature of the carbopol might be enhanced dermal residence time and occlusive nature [29,38,57,58].

The skin penetration profile and drug accumulation of lipid nanoparticle hydrogel formulations in skin tissue were higher than for the RP-C formulation. In addition, in the ex vivo skin permeation experiments, the permeation rate and flux of the lipid nanoparticle enriched hydrogel (due to particle size) was higher than that of RP. Taken together, we hypothesize that drug infiltration into the skin tissue is enhanced for particles in the size range of approximately below 200 nm, in comparison with drugs with micron range in particle size, and this increase in drug infiltration may cause the high skin penetration and drug accumulation in the skin tissue for the nano hydrogel formulations. In contrast to the results in skin penetration and drug accumulation in skin tissue, the absorption rate following the administration of the lipid nanoparticle hydrogel formulations was lower than that following the administration of the RP-C. It could probably be due to the fact that drug solubility can be expected to be enhanced at particle sizes less than 200 nm. From these results, it is possible that the solubility of the drug in the lipid nanoparticle hydrogel formulations are higher than in the RP-C in skin tissue. It is hypothesized that solid drugs that infiltrate into the skin tissue are dissolved, and that the liquid or drugs with higher particle size can then shift into the blood.

#### 3.7.3. PCD Studies

PCD studies were conducted in haloperidol induced PD Wistar rats. Preliminary studies were conducted to confirm the motor impairment of rats after 1 mg/kg IP injection of haloperidol. This dose of the haloperidol could induce the moderate degree of catalepsy and motor imbalance. Standard bar and rotarod tests were used to confirm the induction of PD in the rats. Even though the specific cause of PD still remains an unknown, indications recommend that vast oxidative stress, free-radical formation, genetic susceptibility, and programmed cell death all have a role in the development of PD. The neuropathology of the ailment is based on cell loss in the dopaminergic nigrostriatal tract of the brain, with the corresponding decrease in the striatal DA levels. Formulations were administered as per the experimental procedure to the animal groups. Animals were sacrificed 1 h after administration of haloperidol, brain tissue was collected, and PMS was prepared with striatum. GSH, TBARS, catalase activity and DA levels were estimated.

##### Lipid Peroxidation Levels

**Figure 7:**
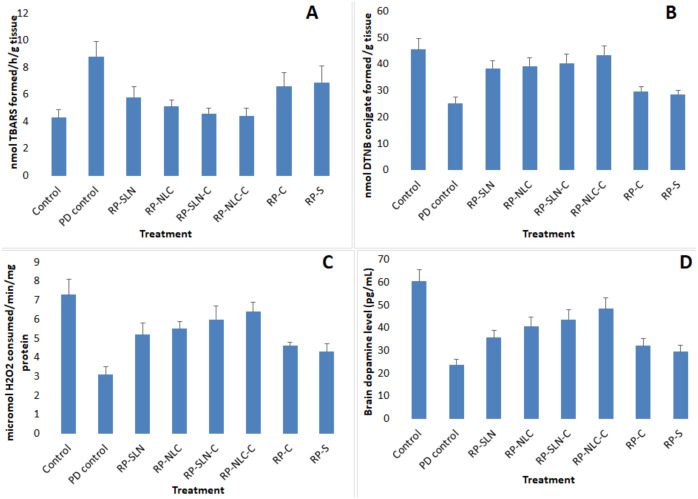
Effect of RP loaded lipid nanoparticles, hydrogel formulations and control formulations on (**A**) thiobarbituric acid reactive substances, (**B**) GSH, (**C**) catalase activity and (**D**) dopamine levels in haloperidol induced Parkinson disease rats (mean ± SD, *n* = 6).

The lipid peroxidation levels of RP in the PMS were estimated as the TBARS concertation (nmol formed/h/g tissue) and the results were shown in Figure 7A. The content of TBARS in the striatum was elevated (104.6%) significantly (*p* < 0.05) in the PD group, as compared to the control group. The increased TBARS level was significantly (*p* < 0.05) restored from RP-SLN and RP-NLC formulation at 48.8% and 62.7%, respectively, compared with PD group. Further, from RP-SLN-C and RP-NLC-C formulation, 76.7% and 88.3% were restored compared with PD group. However, 39.5% and 30.2% levels were restored from RP-C and RP-S, treated groups respectively. Overall, lipid nanoformulations and corresponding hydrogel formulations showed a significant restoration of lipid peroxides than control formulations in PD rats.

##### GSH Levels

GSH levels of the various treated groups were analyzed as nmol DTNB conjugate formed/h/g tissue. From the results, it was observed that the GSH levels were significantly (*p* < 0.05) depleted in the PD group (44.7%) compared to the control group. In case of formulation treated groups, depleted GSH levels were restored as follows: RP-NLC-C (39.9%), RP-SLN-C (33.1%), RP-NLC (30.7%), RP-SLN (28.9%), RP-C (10.1%) and RP-S (7.5%), compared with the PD group. The GSH levels estimated from different groups are shown in Figure 7B.

##### Catalase Activity

Catalase activity of PMS tissue was expressed as micromole H_2_O_2_ consumed/min/mg and the results are presented in Figure 7C. The activity of catalase in the striatum was significantly (*p* < 0.05) decreased (57.5%) in the PD group, as compared to the control group. The depleted level was restored significantly in the treated groups, following the order: RP-NLC-C (45.2%), RP-SLN-C (39.7%), RP-NLC (32.9%), RP-SLN (28.8%), RP-C (20.5%) and RP-S (16.4%), compared with the PD group.

##### DA Levels

Bran DA levels in the striatum were measured (pg/mL) and reported in Figure 7D. DA levels were significantly depleted in D group (60.7%) compared with the control group. The level of dopamine in the treatment group was restored for RP-NLC-C (40.8%), RP-SLN-C (32.9%), RP-NLC (27.9%), RP-SLN (19.8%), RP-C (13.9%) and RP-S (9.6%), compared with PD group.

An inverse relationship has also been previously reported between lipid peroxide and GSH activities and its related enzymes in PD [2,4]. A reduction in GSH may impair H_2_O_2_ clearance and promote OH formation, thus increasing the free radical load, which triggers oxidative stress and consequently disrupts homeostasis. It is reasonable to infer that depletion in GSH triggers lipid peroxide, leading to the degeneration of nigrostriatal neurons, which, in turn, would deplete DA [4]. The increase in the content of GSH and decrease in the extent of lipid peroxide demonstrated the advantageous effects of the developed lipid nanoformulations and corresponding hydrogel formulations in the effective treatment of PD. Similar types of results were also observed with nanoemulsion gel formulation of RP for PD treatment [29].

## 4. Conclusions

The outcome of the present investigation signifies the role of lipid nanoparticles and their enriched hydrogel formulations, considered as an alternative delivery approach and route for the current treatment of PD. RP loaded lipid nanoparticles and hydrogel formulations were successfully developed and optimized. RP-SLN and RP-NLC formulations were stable over three months at different storage conditions and easily manufactured. In vitro release and ex vivo permeation studies confirmed the sustained and prolonged release of the RP and enhanced permeability. PK studies confirmed the improved oral and transdermal bioavailabilities from lipid nanoparticles and hydrogel formulations, respectively. PK studies showed 2.1 and 2.7-folds enhancement from RP-SLN and RP-NLC from oral administration, 3.0 and 3.3-folds enhancement from RP-SLN-C and RP-NLC-C topical administration, compared with control formulations, respectively. RP-SLN-C and RP-NLC-C showed 1.4 and 1.2-folds topical bioavailability enhancement, compared with RP-SLN and RP-NLC oral administration, respectively. PCD studies prove the restoration of biochemical changes in PD model in rats. Therefore, the results demonstrated that SLN, NLC, SLN and NLC enriched hydrogel formulations were considered as alternative approaches and routes for the oral delivery of RP for parkinsonism.

## Figures and Tables

**Table 1:** Composition of ropinirole loaded solid lipid nanoparticles (SLN), nanostructured lipid carriers (NLC), hydrogel and control formulations.

Ingredients (% *w/v*)	RP-SLN	RP-NLC	RP-SLN-C	RP-NLC-C	RP-C	RP-S
RP	0.2	0.2	0.2	0.2	0.2	0.2
Dynasan-114	2.0	1.25	2.0	1.25	-	-
Caproyl 90	-	0.75	-	0.75	-	-
Soylecithin	1.5	1.5	1.5	1.5	-	-
Poloxamer 188	1	1	1	1	-	-
Cabopol 934	-	-	1	1	1	-
Tween 20	-	-	-	-	-	1
Water (mL)	10	10	10	10	10	10

**Table 2:** Particle size, Polydispersity Index (PDI), zeta potential (ZP), assay, drug loading and entrapment efficiency ropinirole loaded SLN and NLC formulations (mean ± SD, *n* = 3).

Formulation	Size	PDI	ZP (mV)	Assay (%)	Drug Loading (DL) (%)	Entrapment Efficiency (EE) (%)
RP-SLN	210.6 ± 3.6	0.21 ± 0.06	−28.4 ± 0.9	97.5 ± 1.3	7.7 ± 0.5	76.8 ± 2.7
RP-NLC	193.2 ± 2.1	0.18 ± 0.03	−30.5 ± 1.5	98.3 ± 2.0	8.4 ± 0.3	84.1 ± 2.3

**Table 3:** pH, viscosity, drug content, spreadability and extrudability of RP-SLN-C and RP-NLC-C hydrogel formulations (mean ± SD, *n* = 3).

Parameter	Formulation	
	RP-SLN-C	RP-NLC-C
pH	6.6 ± 0.3	6.5 ± 0.4
Viscosity (cP)	2678 ± 64.6	2352 ± 77.1
Drug content	98.2 ± 2.1	99.6 ± 2.4
Spreadability (g cm/sec)	6.3 ± 0.5	6.1 ± 0.2
Extrudability (g/cm^2^)	1.4 ± 0.3	1.2 ± 0.2

**Table 4:** Regression coefficient values of RP-SLN, RP-NLC, RP-SLN-C and RP-NLC-C formulations from release kinetic mathematical models.

Formulation	Regression Coefficient (*r*^2^)				
	In Vitro Release				
	Zero Order	First Order	Higuchi	Korsemeyer-Peppas	
				R^2^	n Value
RP-SLN	0.974	0.957	0.960	0.985	0.559
RP-NLC	0.963	0.936	0.970	0.988	0.540
RP-SLN-C	0.977	0.984	0.964	0.993	0.610
RP-NLC-C	0.966	0.974	0.972	0.989	0.546
	**Ex Vivo Permeation**				
RP-SLN	0.980	0.970	0.949	0.995	0.686
RP-NLC	0.968	0.985	0.979	0.999	0.668
RP-SLN-C	0.988	0.990	0.947	0.994	0.706
RP-NLC-C	0.977	0.987	0.958	0.993	0.661

**Table 5:** Ex vivo skin permeation parameters of RP-SLN, RP-NLC, RP-SLN-C, RP-NLC-C and RP-C formulations through rat skin (mean ± SD, *n* = 3).

Formulation	Amount Permeated (µg/cm^2^)	Percentage Drug Permeation at End of 24 h	Flux (µg/cm^2^/h)	Permeability (Percent/cm^2^/h)
RP-SLN	739.2 ± 34.1 *	73.9 ± 3.1 *	29.0 *	2.9 *
RP-NLC	820.7 ± 57.2 *	82.0 ± 2.9 *	32.8 *	3.2 *
RP-SLN-C	561.5 ± 28.9 *	56.1 ± 3.6 *	22.4 *	2.2 *
RP-NLC-C	603.2 ± 41.7 *	60.3 ± 4.2 *	23.9 *	2.3 *
RP-C	296.2 ± 23.5	29.6 ± 3.2	12.9	1.2

* indicates statistically significant at value of *p* < 0.05 compared with RP-C formulation.

**Table 6:** Effect of storage conditions on particle size, PDI, ZP, assay, and entrapment efficiency of optimized RP-SLN and RP-NLC formulation at refrigerator and room temperature (mean ± SD, *n* = 3).

Formulation	Time (Days)	Size (nm)	PDI	ZP (mV)	Assay (%)	EE (%)
		4 °C				
RP-SLN	1	211.5 ± 2.8	0.22 ± 0.04	−29.6 ± 1.2	98.0 ± 1.7	77.5 ± 2.1
	90	220.7 ± 4.7	0.23 ± 0.03	−26.8 ± 1.5	95.8 ± 2.4	74.7 ± 3.2
RP-NLC	1	192.7 ± 2.7	0.17 ± 0.04	−31.6 ± 1.8	99.3 ± 2.2	85.6 ± 1.9
	90	206.4 ± 3.6	0.19 ± 0.05	−28.4 ± 1.6	96.9 ± 2.4	82.4 ± 2.6
		**25 °C**				
RP-SLN	1	211.5 ± 2.8	0.22 ± 0.04	−29.6 ± 1.2	98.0 ± 1.7	77.5 ± 2.1
	90	227.8 ± 3.5	0.24 ± 0.08	−25.2 ± 1.3	94.1 ± 3.0	73.4 ± 2.4
RP-NLC	1	192.7 ± 2.7	0.17 ± 0.04	−31.6 ± 1.8	99.3 ± 2.2	85.6 ± 1.9
	90	210.6 ± 5.3	0.19 ± 0.04	−27.2 ± 2.7	95.8 ± 1.7	81.6 ± 3.1

**Table 7:** Effect of storage conditions on pH, viscosity, assay, spreadability, and extrudability of optimized RP-SLN-C and RP-NLC-C formulation at refrigerator and room temperature (mean ± SD, *n* = 3).

Parameter	RP-SLN-C			
	4 °C		25 °C	
	Day 1	Day 90	Day 1	Day 90
pH	6.5 ± 0.4	6.4 ± 0.5	6.5 ± 0.4	6.2 ± 0.3
Viscosity (cP)	2688 ± 72.5	2703 ± 83.7	2688 ± 72.5	2734 ± 69.8
Drug content (%)	97.9 ± 1.8	96.2 ± 3.6	97.9 ± 1.8	95.8 ± 3.1
Spreadability (g cm/s)	6.4 ± 0.4	6.2 ± 0.3	6.4 ± 0.4	6.1 ± 0.6
Extrudability (g/cm^2^)	1.5 ± 0.4	1.3 ± 0.2	1.5 ± 0.4	1.6 ± 0.2
	**RP-NLC-C**			
pH	6.5 ± 0.4	6.3 ± 0.3	6.5 ± 0.4	6.1 ± 0.3
Viscosity (cP)	2372 ± 69.0	2389 ± 80.5	2372 ± 69.0	2422 ± 66.8
Drug content (%)	99.1± 2.5	97.2 ± 2.1	99.1± 2.5	96.1 ± 3.6
Spreadability (g cm/s)	6.2 ± 0.3	6.3 ± 0.4	6.2 ± 0.3	6.2 ± 0.5
Extrudability (g/cm^2^)	1.3 ± 0.1	1.2 ± 0.4	1.3 ± 0.1	1.4 ± 0.3

**Table 8:** Effect of lyophilization on physicochemical characteristics of RP-SLN and RP-NLC formulations (mean ± SD, *n* = 3).

Parameter	RP-SLN		RP-NLC	
	Pre-Lyophilization	Post-Lyophilization	Pre-Lyophilization	Post-Lyophilization
Size (nm)	224.5 ± 4.2	479.7 ± 8.8	205.4 ± 3.1	434.6 ± 2.8
PDI	0.22 ± 0.05	0.35 ± 0.08	0.21 ± 0.04	0.33 ± 0.05
ZP (mV)	−29.3 ± 1.2	−28.3 ± 2.4	−31.7 ± 2.4	−29.6 ± 0.6
Assay (%)	98.2 ± 1.7	96.4 ± 2.1	99.3 ± 3.1	98.4 ± 1.4
EE (%)	77.5 ± 1.9	75.2 ± 1.5	83.5 ± 3.4	83.6 ± 1.8

**Table 9:** Effect of RP-SLN-C, RP-NLC-C and RP-C formulations on rat skin irritation after topical administration (*n* = 4).

Group	Erythema		Edema	
	24	48	24	48
I (negative control)	0	0	0	0
II (RP-C)	0	1	0	1
III (RP-SLN-C)	0	0	0	0
IV (RP-NLC-C)	0	0	0	0
V (Formalin)	2	3	3	3

Erythema scale: 0 = none, 1 = slight, 2 = well-defined, 3 = moderate, and 4 = scar formation. Edema scale: 0 = none, 1 = slight, 2 = well-defined, 3 = moderate, and 4 = severe.

**Table 10:** Pharmacokinetic parameters of RP from RP-SLN, RP-NLC, RP-S and RP-SLN-C, RP-NLC-C, RP-C formulations after single dose oral and transdermal administration, respectively, in Wistar rats (mean ± SD, *n* = 6).

Parameter	RP-SLN	RP-NLC	RP-SLN-C	RP-NLC-C	RP-C	RP-S
***C*_max_ (µg/mL)**	7.1 ± 0.9 *	8.3 ± 0.7 *	8.1 ± 1.7 ^#^	8.9 ± 1.4 ^#^	6.1 ± 1.3	5.9 ± 1.2
***t*_max_ (h)**	4.0 ± 0.0 *	4.0 ± 0.0 *	6 ± 0.0 ^#^	6 ± 0.0 ^#^	3 ± 0 ^@^	1 ± 0
***AUC*_tot_ (µg/mL·h)**	49.8 ± 5.8 *	62.7 ± 4.2 *	69.8 ± 5.6 ^#, $^	76.8 ± 4.8 ^#, $^	34.8 ± 2.9 ^@^	22. 8 ± 2.4
***t*_half_ (h)**	10.1 ± 1.6 *	10.3 ± 1.0 *	10.4 ± 1.6 ^#^	11.6 ± 0.9 ^#^	7.1 ± 1.3	6.7 ± 1.1
**MRT (h)**	12.9 ± 1.3 *	13.2 ± 1.7 *	14 ± 2 ^#^	15.3 ± 2.5 ^#^	7.6 ± 1.1	5.5 ± 0.9

* indicates statistically significant at *p* < 0.05 level compared with RP-S formulation; ^#^ indicates statistically significant at *p* < 0.05 level compared with RP-C control formulation; ^$^ indicates statistically significant at *p* < 0.05 level compared with RP-SLN and RP-NLC formulation and ^@^ indicates statistically significant ap *p* < 0.05 compared with RP-S formulation.
